# *Herbaspirillum* Infection in Humans: A Case Report and Review of Literature

**DOI:** 10.1155/2020/9545243

**Published:** 2020-02-19

**Authors:** Rashmi Dhital, Anish Paudel, Nidrit Bohra, Ann K. Shin

**Affiliations:** Reading Hospital and Medical Center, Tower Health System, West Reading, PA, USA

## Abstract

*Introduction*. *Herbaspirillum seropedicae* are Gram-negative oxidase-positive nonfermenting rods of Betaproteobacteria class, commonly found in rhizosphere. More recently, some *Herbaspirillium* species have transitioned from environment to human hosts, mostly as opportunistic (pathogenic) bacteria. We present a 58-year-old female with non-small-cell lung cancer (NSCLC) who presented with pneumonia and was found to have *Herbaspirillum seropedicae* bacteremia. *Case History*. A 58-year-old woman with NSCLC on Pralsetinib presented with fevers and rigors for 2 days. Coarse breath sounds were auscultated on the right upper lung field. Labs revealed leukopenia and mild neutropenia. CT chest revealed right upper lobe pneumonia. She was admitted for sepsis secondary to pneumonia and placed on broad spectrum antibiotics with intravenous piperacillin-tazobactam and vancomycin. The patient continued to have fever 2 days after admission (max: 102.8°F). Preliminary blood cultures grew Gram-negative rods. The patient continued to have temperature spikes on the 3rd day of antibiotics (*T*_max_ 101.5°F). Blood cultures revealed oxidase-positive nonfermenting rods. The patient's antibiotic was changed to IV meropenem on the 4th day of hospitalization. Ultimately, on the seventh day of hospitalization, the blood culture was confirmed from outside lab as *Herbaspirillum seropedicae*. The patient started feeling better and defervesced after about 24 hours. *Discussion*. More recently, *Herbaspirillum spp*. have been recovered from humans. Our patient had *Herbaspirillum* bacteremia, and reported regularly cleaning her pond and weeding her garden with possible exposure to this environmental proteobacterium. *Herbaspirillum* may be more prevalent than earlier thought owing to misidentification. With the institution of appropriate antimicrobial therapy, the outcomes seem mostly favorable.

## 1. Introduction

*Herbaspirillum seropedicae* are Gram-negative oxidase-positive nonfermenting rods of Betaproteobacteria class, commonly found in the rhizosphere of maize and rice, which promote plant growth via biological nitrogen fixation and phytohormone production [1, 2]. More recently, some *Herbaspirillium* species have transitioned from environment (water, contaminated soil, plant internal tissues, and root nodules) to human hosts, mostly as opportunistic (pathogenic) bacteria. Cases of human colonization and infection have mostly been noted in cystic fibrosis and immunocompromised cancer patients [3–8] and also in some patients without an apparent immunosuppressed state [9–11].

### 1.1. Case History

A 58-year-old woman with a medical history of non-small-cell lung cancer (NSCLC) with malignant left pleural effusion (with RET mutation under phase 1 trial with pralsetinib), nonbacterial thrombotic endocarditis, and pulmonary embolism presented to the hospital with fevers, chills, and rigors for 2 days. At presentation, she was febrile with a temperature of 101 F and tachycardic at 114 beats/minute. Physical examination revealed a Port-A-catheter on the right upper chest. Coarse breath sounds were auscultated on the right upper lung field. Labs revealed anemia with the hemoglobin level of 6.4 (ref: 12.0–16.0 g/dl), leukopenia with the white blood cell (WBC) count of 1.7 (ref 4.8–10.8 10*E*3/ul), mild neutropenia with the absolute neutrophil count (ANC) of 1353 cells per microliter, an elevated C-reactive protein (CRP) level of 22.52 (ref range <1.00) mg/dl, and an elevated creatinine level of 1.56 mg/dl (baseline creatinine of 1 mg/dl). CT chest revealed right upper lobe pneumonia, scattered small pulmonary nodules consistent with metastatic disease, and stable bony metastatic disease of the anterior left 6th rib. She was admitted for sepsis secondary to pneumonia and placed on broad spectrum antibiotics with intravenous piperacillin-tazobactam and vancomycin. She received 2 units of packed red cell transfusion with stable hemoglobin thereafter. Urine analysis was unremarkable. Urine legionella and streptococcus pneumonia antigens were negative.

The patient continued to have fever 2 days after admission (max: 102.8 F). Preliminary blood cultures done at our hospital at Day 2 of hospitalization revealed Gram-negative rods. CT abdomen and pelvis revealed small volume of ascites and jejunal wall thickening with mild adjacent mesenteric edema, suggestive of enteritis. CT chest revealed persistent right upper lobe pneumonia. The patient continued to have temperature spikes on the 3rd day of antibiotics (*T*_max_ 101.5 F). Blood cultures revealed oxidase-positive nonfermenting rods. The organism was not clearly identified in our lab, and the specimen was sent to Central Pennsylvania Alliance Laboratory for identification by DNA sequencing using MALDI (matrix-assisted laser desorption/ionization) technique. The patient's antibiotic was changed to IV meropenem on the 4th day of hospitalization. Port was removed and catheter tip culture was sent, which came out to be negative.

Ultimately, on the seventh day of hospitalization, the blood culture was identified from outside lab as *Herbaspirillum seropedicae* (which was susceptible to all tested antibiotics including amikacin, cefepime, ceftazidime, ciprofloxacin, aztreonam, levofloxacin, meropenem, piperacillin/tazobactam, and ticarcillin/clavulanate). The patient started feeling better and defervesced after about 24 hours of being on meropenem. The patient was discharged with a midline catheter to continue IV meropenem for a total of 14 days and follow-up for outpatient monitoring. The WBC count at discharge was 5.1 × 10 *µ*/L, ANC was 3978 cells/microliter, and CRP was 6.36 mg/dl. On further questioning to assess for risk factors, the patient reported taking care of her pond and regularly cleaning weeds.

## 2. Discussion

The first described species of the genus *Herbaspirillum* was the bacterium *Herbaspirillum seropedicae*, which was found colonizing the roots and aerial parts of important crops [1]. More recently, *Herbaspirillum spp*. have been recovered from the blood and sputum/bronchoalveolar lavage of patients with cystic fibrosis and pneumonia [4, 6, 11] and from blood in patients with leukemia [6, 7, 12], aplastic anemia [7], multiple myeloma [13], and cellulitis [8]. Our patient is a 58-year-old woman with NSCLC on RET-inhibitor pralsetinib who presented with right upper lobe pneumonia and had blood culture positive for oxidase-positive nonfermenting Gram-negative rod, which was confirmed as *Herbaspirillium* only on Day 7 of hospitalization by which time the patient was already improving with meropenem therapy. On retrospect, our patient had been regularly cleaning her pond and weeding her garden with possible exposure to this environmental proteobacterium, supporting this change in dynamics from environmental bacterium to opportunistic pathogen. However, it is not entirely clear why the patient only defervesced and improved after 24 hours of switching piperacillin/tazobactam to meropenem, even though her culture report suggested sensitivity to piperacillin/tazobactam. New genomic data findings suggest that the transition from environmental to pathogenic led to the loss and acquisition of specific genes to allow colonization and survival in new environments. The strains that have been infectious to humans have lost the genes for nitrogen fixation and acquired genes for lipopolysaccharide biosynthesis with the addition of sialic acids to evade the immune system [1].

We reviewed the literature for available *Herbaspirillum* cases and identified 9 published studies from 2005 to 2019 (Table 1), with *Herbaspirillum* bacteremia, cellulitis or pneumonia. Tan and Oehler reported *Herbaspirillum* bacteremia and cellulitis in a patient with aquatic exposure [8]. Ziga et al. reported *Herbaspirillum* bacteremia in a 2-year-old girl with a history of acute lymphoblastic leukemia (ALL) after induction chemotherapy and stem cell transplant [6]. Chen et al. reported *Herbaspirillum* bacteremia in an ALL patient on chemotherapy after taking sugarcane juice [12]. Regunath et al. described a case of bacteremia caused by gentamicin-resistant *Herbaspirillum* in an immunocompetent adult male farmer [11]. Suwantarat et al. reported the first fatal case-related *H. seropedicae* bacteremia secondary to pneumonia in an immunocompromised 65-year-old man with end-stage renal disease and multiple myeloma [13]. Abreu-Di Berardino et al. described *Herbaspirillum huttiense* pneumonia in a patient with essential thrombocytosis [9]. Liu et al. reported *Herbaspirillum huttiense* bacteremia in an elderly patient with no obvious immune suppression, who later went on to develop a pneumonia, where despite adequate treatment, microbiological eradication was not easily achieved, and septicemia lasted for several days along with sputum culture positivity for *Herbaspirillum huttiense* 2 months later [10].

Chemaly et al. investigated a potential cluster of hospital-based *Herbaspirillum* infections in cancer patients at the University of Texas MD Anderson Cancer Center, initially identified as *Burkholderia cepacia* complex and subsequently reidentified as *Herbaspirillum* species by the Cystic Fibrosis Foundation *Burkholderia cepacia* Research Laboratory and Repository (BcRLR) at the University of Michigan. The authors identified a total of 8 patients with bacteremia and pneumonia with cultures positive for *Herbaspirillum* species between July 2011 and August 2012 (including 5 clusters and 3 additional cases identified prospectively). 5 of the 8 patients were females and the median age was 53 years (2–67 years) [7].

Spilker et al. reported a 26-year-old male with moderate to severe lung disease with Gram-negative rod bacteremia at hospital day 23. The patient was admitted for methicillin-resistant *staphylococcus aureus* (MRSA) and *Pseudomonas* pneumonia, and cultures were initially misidentified as *Burkholderia cepacia* complex [4]. The authors analyzed isolates from sputum from over 1,100 cystic fibrosis patients across 8 years (January 2000 to December 2007) and found *Herbaspirillum* in only 28 patients (<3%) with ages ranging from 20 months to 5 years, of which 3 isolates were *Herbaspirillum huttiense*, 3 were *Herbaspirillum frisingense*, 2 were *Herbaspirillum seropedicae*, and 2 were *Herbaspirillum putei*, and the remaining eighteen isolates could not be speciated [4]. Prior to the correct identification of *Herbaspirillum* at the BcRLR for the above 28 specimen, they had been identified as *Burkholderia cepacia* complex in 19 (68%) and *Ralstonia* in 4 (14%). Most patients appeared to have had transient respiratory tract colonization with *Herbaspirillum* except for bacteremia in one patient and a chronic respiratory tract infection in another [4].

Tetz and Tetz, for the first time, identified *Herbaspirillum frisingense* from the bladder of a human patient with urinary tract infection (UTI). Furthermore, genome analysis revealed numerous factors such as adhesins, urease, hemolysin D, and pilin that contribute to the bacterium's virulence in UTIs. The authors suggested that further research of this *Herbaspirillum* spp would aid in better understanding of its implication in UTI [14]. Similarly, Wu et al. performed a comprehensive analysis of urinary microenvironment of bladder cancer and identified *Herbaspirillum* as one of the species associated with an increased risk of progression, suggesting its potential role in risk stratifying bladder cancer [15].

As evidenced by the above studies, *Herbaspirillum* is an emerging pathogen and may be more prevalent than earlier thought owing to misidentification for organisms like *Burkholderia cepacia* complex due to phylogenetic and phenotypic resemblance [4, 6, 7, 13]. Antimicrobial susceptibilities may serve as a means for differentiating *Herbaspirillum* species from *Burkholderia cepacia* complex because the latter are usually multidrug resistant, whereas *Herbaspirillum* is not. With the institution of appropriate antimicrobial therapy, the outcomes seem mostly favorable and the bacteria appear to be easily eradicated, except in 1 case reported by Liu et al. Misidentification as *Burkholderia* complex can have serious implications for clinical care, and distinction between these is important due to different resistance profiles and different therapeutic implications. The increased availability of newer molecular methods (e.g., MALDI-TOF MS) should allow laboratories to correctly identify this organism and reduce misidentification by established microbial identification systems.

## Figures and Tables

**Table 1:** Summary table of published cases of *Herbaspirillum* infection in humans.

Author year, country	Age/sex	Past history/predisposing conditions/cancer/HCST	Immune suppression	Likely risk factor/inciting event	Reason for admission/Clinical presentation	Initial antibiotics	Response to initial antibiotics/subsequent antibiotics	Specimen source positive for *Herbaspirillum*
Liu 2019, Korea	93/F	Hypertension Advanced age	N/A	—	(i) Fever, seizure (ii) After a few days, hypoxia and chest X-ray with pneumonia	Empiric vancomycin + ceftriaxone for encephalitis	Changed to meropenem and colistin at 10 days and changed to ceftazidime, minocycline, and trimethoprim/sulfamethoxazole thereafter	Blood: *Herbaspirillum huttiense* 2 months later, sputum: *Herbaspirillum huttiense*
Abreu-di berardino 2019, Spain	59/F	Aortic wall thrombosis, visceral and cerebral ischemic lesions, JAK2 + essential thrombocytopenia, new DM	N/A	—	(i) Generalized deconditioning (ii) Dyspnea 10 days after admission, nosocomial pneumonia	Piperacillin-tazobactam	Recovered completely after antibiotics	Sputum: *Herbaspirillum huttiense*
Chen 2010, China	48/F	Acute lymphoblastic leukemia	On chemotherapy and G-CSF	Drank sugarcane juice before fever started	Fever, chills	Cefmetazole and gatifloxacin	Improved	Blood: *Herbaspirillum huttiense*
Spilker 2008, USA	26/m	Moderate to severe lung disease, pancreatic insufficiency, diabetes, and liver disease	Recent multiple admissions for exacerbation of respiratory symptoms	—	Fevers and rigors, MRSA, and *Pseudomonas aeruginosa* bacteremia	Vancomycin, piperacillin-tazobactam, and tobramyicn *x* 20 days Antibiotic discontinued at 20 days, after FEV1 started to improve	On the hospital day 23: fever and rigors Changed to intravenous ceftazidime and tobramycin and oral trimethoprim-sulfamethoxazole (TMP-SMX), levofloxacin, and minocycline	Blood: (GNR, initially identified as *burkholderia cepacia* complex) Later, identified as *Herbaspirillum species*
Tan 2005, USA	49/m	Probable hepatic cirrhosis	—	Homeless Jumped from a bridge into a freshwater canal in central Florida	Increasing erythema and warmth to the left leg (cellulitis)	Ampicillin/sulbactam	Antibiotics switched to cefepime and levofloxacin after initial blood culture results	Blood: (oxidase-positive nonlactose-fermenting GNR,submitted to an outside reference laboratory) positive for *Herbaspirillum seropedicae*
Regunath 2014, USA	46/M	Childhood asthma, atypical pneumonia as a teenager, tonsillectomy	N/A	Farming in rural Missouri, close contact with cattle and turkeys, mold and possible rat excreta Drenched in rain during a fishing trip	Fever, fatigue, SOB, night sweats, anorexia, myalgia, and headache Dry cough, right-sided pleuritic chest pain, and worsening dyspnea Hypoxia (multilobar pneumonia)	Vancomycin, ceftriaxone, and azithromycin	Ceftriaxone switched to piperacillin-tazobactam Azithromycin switched to Doxycycline After 12 days of treatment, the patient improved and was extubated	Blood (Day 1) (from referring facility) identified as *Burkholderia cepacia* complex (BCC),later as *Herbaspirillum aquaticum* or *Herbaspirillum huttiense* BAL (Day 3): GNR as *Herbaspirillum aquaticum* or *Herbaspirillum huttiense*
Chemaly 2015, USA								
Hospital-based cluster of *Herbaspirillium sp* infections initially misidentified as *B. cepacia*	48/F	Ovarian adenocarcinoma	Chemotherapy	Source and mechanism of the cluster unknown	Pseudomonas BSI, sepsis-CRBSI	Cefepime (5 patients), ceftazidime (1 patient), moxifloxacin (1 patient), meropenem (1 patient) initially Followed by ceftriaxone or fluoroquinolone	All patients improved with antibiotic and had negative repeat blood cultures 1 patient had recurrence, which resolved once the port was removed	Blood, Infusaport tip
	67/F	Leukemia	Chemotherapy		MRSA pneumonia, sepsis-BSI			Blood
	58/M	Leukemia/HSCT	High-dose steroid		GI bleed GVHD, BSI			Blood
	55/F	Leukemia/HSCT	High-dose steroid		GI GVHD, BSI			Blood
	2/M	Ependymoma	High-dose steroid		*Herbaspirillium* Sepsis-BSI			Blood
3 additional *Herbaspirillium* sp after continued surveillance (*sporadic*)	66/F	History of recurrent pneumonia, lung cancer	Radiation therapy		*Herbaspirillium* Sepsis-pneumonia			Sputum
	18/M	Lymphoma	Chemotherapy		Chemotherapy Sepsis-BSI			Blood
	51/F	Aplastic anemia/HSCT	Tacrolimus		*Herbaspirillium* Sepsis- CRBSI			Blood, PICC line tip
Suwantarat 2015, USA	65/M	Multiple myeloma ESRD on hemodialysis	Steroids and lenalidomide		Acute respiratory failure and septic shock Right lower lobe pneumonia	Vancomycin, cefepime, ciprofloxacin, and micafungin	Changed to vancomycin, meropenem, and gentamicin within 12 hours However, the patient remained clinically unstable and died after 4 days	BAL (oxidase-positive, nonlactose-fermenting GNR), initially identified as *Cupriavidus*, later as *Herbaspirillum* Blood: *Herbaspirillum seropedicae*
Ziga 2010, USA	2/F	Acute lymphoblastic leukemia	Chemotherapy, HSCT	Uncertain Lives with her parents and grandparents on a large farm	Fever and diarrhea	Cefepime	Gentamicin added after oxidase-positive, weakly catalase-positive, Gram-negative bacillus Later, switched to meropenem	Blood *Burkholderia cepacia* complex As susceptibility pattern was not consistent, further identification was pursued: *Herbaspirillum* sp

G-CSF: granulocyte-colony stimulating factor; BAL: bronchoalveolar lavage; GI GVHD: gastrointestinal graft versus host disease; BSI: blood stream infection; CRBSI: catheter-related blood stream infection; HSCT: hematopoietic stem cell infection; GNR: gram-negative rod.
